# Clinical and Angiographic Outcomes after Intracoronary Bare-Metal Stenting

**DOI:** 10.1371/journal.pone.0094319

**Published:** 2014-04-11

**Authors:** I-Chang Hsieh, Ming-Jer Hsieh, Shang-Hung Chang, Chao-Yung Wang, Cheng-Hung Lee, Fen-Chiung Lin, Chun-Chi Chen

**Affiliations:** 1 Second Department of Cardiology, Percutaneous Coronary Intervention Center, Chang Gung Memorial Hospital, Chang Gung University College of Medicine, Linkou, Taoyuan, Taiwan; 2 Graduate Institute of Clinical Medical Sciences, Chang Gung University, Taoyuan, Taiwan; S.G.Battista Hospital, Italy

## Abstract

**Background:**

Data from a large patient population regarding very long-term outcomes after BMS implantation are inadequate. This study aimed to evaluate the very long-term (8–17 years) clinical and long-term (3–5 years) angiographic outcomes after intracoronary bare-metal stenting (BMS).

**Methods and Results:**

From the Cardiovascular Atherosclerosis and Percutaneous TrAnsluminal INterventions (CAPTAIN) registry, a total of 2391 patients with 2966 lesions treated with 3190 BMSs between November 1995 and May 2004 were evaluated. In total, 1898 patients with 2364 lesions, and 699 patients with 861 lesions underwent 6-month and 3- to 5- year angiographic follow-up, respectively. During a mean follow-up period of 149±51 months, 18.6% of the patients died (including 10.8% due to cardiac death), 6.1% developed reinfarction, 16.2% had target lesion revascularization (including 81% of the patients within the first year), 14.5% underwent new lesion stenting (including 72% of the patients after 3 years), 2.4% underwent coronary bypass surgery, and 1.6% had definite stent thrombosis. The overall cardiovascular event-free survival rate was 58.5%. The 6-month angiographic study indicated a 20% restenosis rate. The minimal luminal diameter increased from 0.65±0.44 mm to 3.02±0.46 mm immediately after stenting, decreased to 2.06±0.77 mm at the 6-month follow-up, and increased to 2.27±0.68 mm at the 3- to 5-year follow-up.

**Conclusions:**

This study provides clinical and angiographic results from a large population of patients who underwent BMS implantations after a long-term follow-up period (149±51 months). The progression of coronary atherosclerosis developed over time, and presented with new lesion required stent implantation. The follow-up angiographic findings reconfirmed the late and sustained improvement in luminal diameter between 6 months and 3–5 years.

## Introduction

Percutaneous coronary intervention (PCI) with bare-metal stents (BMSs) has been shown to reduce procedural complications and late restenosis compared with balloon angioplasty in randomized trials [1], [2]. Compared with BMSs, further development of drug-eluting stents (DESs) can reduce in-stent restenosis and the need for repeated revascularization [3], [4]. Nevertheless, BMSs are still used in PCI, especially for simple lesions, despite the superiority of DES. The permanent placement of metallic prosthetic devices has benefits, however, the long-term efficacy and safety remains controversial. Several studies have shown bare-metal stenting to be beneficial in mid-term [5]–[7], long-term [8]–[10] and very long-term follow-up [11]. Kimura et al. [12], [13] reported a triphasic luminal response characterized by an early restenosis phase until 6 months, an intermediate-term regression phase from 6 months to 3 years, and a late re-narrowing phase beyond 4 years. However, only a small number of patients were included in these studies. Therefore, this study aimed to evaluate the very long-term (8–17 years) clinical and long-term (3–5 years) angiographic outcomes of a large sample (2391 consecutive patients) who underwent BMS implantation, and identify specific predictors of clinical cardiovascular events and 6-month angiographic in-stent restenosis.

## Materials and Methods

### Patient population

The Cardiovascular Atherosclerosis and Percutaneous TrAnsluminal INterventions (CAPTAIN) registry is a prospective, physician-initiated, single-center observational database that has been maintained since November 1995. This is an ongoing registry and includes the data of 6200 consecutive patients who underwent elective or emergent PCI with stenting at our hospital from November 1995 to July 2013. In this study, we enrolled 2391 consecutive patients from the CAPTAIN registry who underwent BMS implantation between November 1995 and May 2004. Ethical approval of this study was obtained from the Institutional Review Board of Chang Gung Medical Foundation. All the patients provided informed consent to undergo the procedure and follow-up protocol. All the participants in this manuscript has given written informed consent to publish these case detail. The inclusion criteria for stenting were as follows: evidence of myocardial ischemia and >50% stenosis in a native coronary artery or in a bypass vein graft that was suitable for stenting. The exclusion criteria were as follows: severe multivessel disease requiring bypass surgery, contraindication for aspirin, ticlopidine or clopidogrel use, and patients who refused to undergo the procedure. Dual antiplatelet therapy with aspirin and ticlopidine/clopidogrel was administered to all of the patients for at least 1 month. The enrolled patients were stratified into three groups according to the presentation of ST-segment elevation myocardial infarction (STEMI, 1020 patients), non-ST-segment elevation myocardial infarction (NSTEMI, 206 patients), and non-myocardial infarction (non-MI, 1165 patients). We performed an analysis of immediate and late outcomes among these three groups.

### Interventional procedure and clinical follow-up

The procedure of stent implantation was performed through the femoral or radial artery according to standard techniques. A predilation was performed using an undersized balloon if the lesion was very tight (>70% stenosis). The choice of the type of stent to be implanted was made by the operator, mainly on the basis of the available stent size. After initial stent deployment, high-pressure balloon inflation (≥14 atm) was applied. Cardiac isoenzymes were measured in all of the patients immediately and 6 hours after the procedure. Information on clinical status, medical management, and occurrence of any adverse events was obtained from the patients' medical records. The patients were clinically followed up through outpatient visits or telephone contact. Clinical follow-up was scheduled at 1, 2, 3, 6, 9 and 12 months, and every 3 months thereafter. Angiographic follow-up was recommended as routine after 6 months and 3 years later or earlier in cases of suspected recurrent myocardial ischemia. If the patients developed chest pain with evidence of myocardial ischemia and greater than 50% stenosis in a native coronary artery or a bypass vein graft angiographically, further PCI with stenting was performed if appropriate. Multivariate predictors of long-term cardiovascular event and 6-month angiographic in-stent restenosis were evaluated.

### Angiographic analysis

Quantitative angiographic analysis was conducted with a selected end-diastolic cine-frame that showed stenosis in its most severe and non-foreshortened view. A contrast-filled guiding catheter was used as reference for calibration. Random measurements were performed by two blinded experienced angiographers. The interobserver correlation coefficient (r) was 0.93 (p<0.01), and the intraobserver correlation coefficient was 0.95 (p<0.01). The minimal luminal diameter (MLD), the reference vessel diameter, percentage of diameter stenosis, and balloon diameter were measured by automatic edge-detection or a digital caliber method (DCI or Integris BH3000, Philips, Eindhoven, The Netherlands). Binary restenosis was defined as ≥50% stenosis of the luminal diameter in the target lesion at the time of the follow-up angiography. Acute gain was defined as the difference between the baseline and the final MLD, late loss as the difference between final post-stenting and the follow-up MLD, net gain as the difference between acute gain and late loss, and loss index as the ratio of late loss to acute gain. The left ventricular ejection fraction was measured from the left ventricular angiogram obtained at a right anterior oblique projection with an angle of 30°. The in-stent restenosis patterns were classified as focal (pattern I, ≤10 mm in length), diffuse (pattern II, >10 mm within the stent), proliferative (pattern III, >10 mm extending outside the stent), and totally occluded (pattern IV) [14]. We also evaluated the impact of the SYNTAX score on the long-term patency rate. The SYNTAX (SYNergy between PCI with TAXUS and cardiac surgery) score for the 699 patients who received 3- to 5- year follow-up angiography was calculated retrospectively by scoring all coronary lesions with a diameter stenosis of 50% or more in a vessel diameter of 1.5 mm or more by using the SYNTAX score algorithm. All of these patients were then divided into three groups according to the SYNTAX score: the low score group (0–22), the intermediate score group (23–32), and the high score group (≥33).

### Definitions

We defined an in-hospital major adverse cardiac event (MACE) as death, STEMI or NSTEMI, need for an emergency bypass surgery, or a vascular complication (pseudoaneurysm or arteriovenous fistulae at the access site requiring surgical treatment). The cardiovascular events during the follow-up period included cardiac death, reinfarction (STEMI or NSTEMI), target lesion revascularization (TLR), stenting in a new lesion, necessitation of coronary bypass surgery, or a cerebrovascular accident. STEMI was diagnosed by standard methods if the patient experienced prolonged chest pain (longer than 30 minutes) that could not be relieved by nitroglycerin, showed an ST-segment elevation of ≥0.2 mV in at least two contiguous electrocardiographic leads, and had significantly elevated creatine kinase-MB enzyme levels.

### Statistical analysis

We used STATA statistical software (version 10) for the statistical analyses. The final results were represented as mean±standard deviation or as percentages, and categorical data were presented as numbers. The normality of all the variables was analyzed. To assess determinants of dependent variables, we estimated logistic odds ratios (ORs) with their 95% confidence intervals (CIs). Logistic ORs were estimated in a multivariate analysis performed in the logistic forward stepwise regression model. Independent variables were identified on the basis of the findings of the bivariate analyses. Subjectively, those with “borderline significance” (p<0.10) were included. A p value <0.05 was considered significant.

## Results

### Clinical and angiographic features

**Figure 1:**
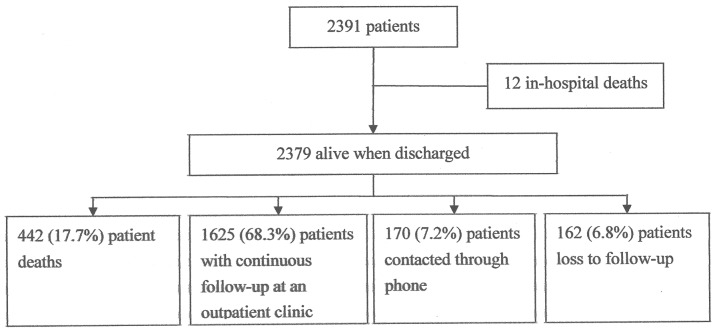
Flow Chart of the Patients During the Long-term Follow-up Period.

A total of 2391 patients with 2966 lesions who underwent 3190 BMS implantations were enrolled in this study. Twelve patients died in the hospital, and 2379 patients survived and were discharged from the hospital. During a long-term mean follow-up period of 149±51 months, 442 patients (17.7%) died, 1625 (68.3%) underwent continuous follow-up at an outpatient clinic, 170 (7.2%) were contacted with telephone, and 162 (6.8%) were lost to follow-up (Fig. 1). The patients' baseline characteristics are listed in Table 1. In total, 2966 lesions were recorded, the majority of which were located at the left anterior descending artery (1456, 49.1%), followed by the right coronary artery (912, 30.7%), and the left circumflex artery (486, 16.4%). Of these lesions, 172 (5.8%), 939 (31.7%), 1514 (51%), and 341 (11.5%) were ostial, proximal, middle, and distal lesions, respectively. Complex lesions (types B2 and C) were noted in 2252 (76%) of the lesions. The lesions had a mean length of 18±9 mm (Table 2).

**Table 1:** Patient Characteristics.

No. of patients	2391
Age(years)	61±11
Male	1945(81.3%)
Hypertension	1148(48.0%)
Diabetes mellitus	579(24.2%)
Smoking	1165(48.7%)
Hyperlipidemia	1474(61.6%)
Family history of CAD	58(2.4%)
CAD	
Left main	47(2.0%)
1-vessel	988(41.3%)
2-vessel	787(32.9%)
3-vessel	569(23.8%)
Previous PTCA	56(2.3%)
Recent infarction	1056(44.2%)
Unstable angina	935(39.1%)
LVEF(%)	56±14

CAD: coronary artery disease; LVEF: left ventricular ejection fraction; PTCA: percutaneous transluminal coronary angioplasty.

**Table 2:** Lesion Characteristics and Procedural Data.

No. of lesions	2966
Target vessel location	
Left main	54(1.8%)
LAD	1456(49.1%)
LCX	486(16.4%)
RCA	912(30.7%)
Diagonal	9(0.3%)
OM	26(0.9%)
Graft	15(0.5%)
Ramus	6(0.2%)
PL	2(0.1%)
Type	
A	74(2.5%)
B1	640(21.6%)
B2	1366(46.1%)
C	886(29.9%)
Restenosis lesions	65(2.2%)
Lesions length (mm)	18±9
Lesions morphology	
Segmental	2544(85.8%)
Eccentric	1441(48.6%)
Calcification	526(17.7%)
Bending ≧45°	234(7.9%)
Thrombus	269(9.1%)
Chronic total occlusion	117(3.9%)
No. of stents	3190
Maximal balloon diameter (mm)	3.42±0.47
Balloon/vessel diameter (mm)	1.10±0.07
Maximal pressure (atm)	14.3±2.2

LAD: left anterior descending coronary artery; LCX: left circumflex coronary artery; RCA: right coronary artery; OM: obtuse marginal artery; PDA: posterior descending artery; PL: posterolateral artery;

### Procedural and in-hospital results

A total of 3190 stents were implanted into the 2966 lesions, including 655 Palmaz-Schatz (Johnson & Johnson, Warren, New Jersey, USA), 428 Multilink (Guidant, Santa Clara, CA, USA), 102 AVE (Medtronic, Minneapolis, MN, USA), 18 Crown (Johnson & Johnson), 293 Duet (Guidant), 123 Crossflex (Johnson & Johnson), 553 Tristar (Guidant), 344 Bx (Johnson & Johnson), 148 S7 (Medtronic), 95 Express (Boston, Natick, Massachusetts, USA), 22 Tetra (Guidant), 326 Penta (Guidant), 44 Pixel (Guidant) and 39 R stents (OrbusNeich, Hong Kong). The balloon-to-vessel diameter ratio was 1.10±0.07, and the mean maximal balloon dilatation pressure was 14.3±2.2 atm (Table 2). Twelve patients (0.5%) died in the hospital, and 48 patients (2%) had procedural myocardial infarctions (0.2% with STEMI and 1.8% with NSTEMI). Three patients (0.1%) underwent emergency bypass surgery, and 6 (0.3%) had vascular complications at the access site. The in-hospital MACE rate was 2.8% (Table 3). There was no difference among the STEMI, NSTEMI and non-MI groups regarding the in-hospital MACE rate (2.75%, 1.94%, and 3.04%, respectively, p = 0.735).

**Table 3:** In-hospital Events.

No. of patients	2391
Death	12(0.5%)
Procedural myocardial infarction	
ST-elevation	4(0.2%)
Non ST-elevation	44(1.8%)
Emergency bypass surgery	3(0.1%)
Pseudoaneurysm at the access site requiring surgical repair	6(0.3%)
Major adverse cardiac events	67(2.8%)

### Angiographic analysis

**Figure 2:**
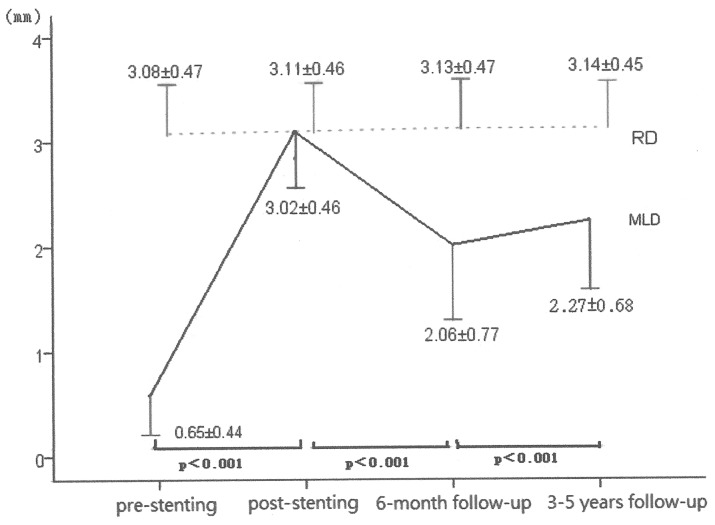
Serial changes of minimal luminal diameter at pre-stenting, post-stenting, 6-month follow-up and 3–5 years follow-up.

A 6-month angiographic follow-up was performed in 1898 patients (80% of the eligible patients) with 2364 lesions 197±60 days after stent implantation. The percentage of diameter stenosis was 80±13% before stenting, 3±6% immediately after stenting, and 36±21% at the 6-month follow-up. The MLD was 0.63±0.43 mm before stenting, 3.04±0.45 mm immediately after stenting, and 2.00±0.77 mm at the 6-month follow-up. The reference vessel diameter did not change significantly and was 3.12±0.47 mm before stenting, 3.13±0.46 mm immediately after stenting, and 3.13±0.45 mm at the 6-month follow-up period. The acute gain was 2.41±0.54 mm, the late loss 1.04±0.67 mm, the loss index 0.45±0.30, and net gain 1.37±0.83 mm. The restenosis rate was 23% per patient and 20% per lesion (Table 4). Among the 471 restenosis lesions, 186 (40%) were classified as pattern I restenosis, 196 (42%) as pattern II, 30 (6%) as pattern III, and 59 (12%) as pattern IV. In the 186 pattern I lesions, balloon angioplasty for in-stent restenosis was performed in 135 (72.5%), and further follow-up angiography (3–5 years after initial stenting or ischemia driven) in 66 lesions, 7 of which developed re-restenosis (10.6%). Of the 196 pattern II lesions, 144 (73.5%) received balloon angioplasty for in-stent restenosis and 68 lesions received further follow-up angiography, of which 15 developed re-restenosis (22%). Of the 30 pattern III lesions, 27 (90%) received balloon angioplasty and 12 received further follow-up angiography, of which 3 developed re-restenosis (25%). Of the 59 pattern IV lesions, 2 received balloon angioplasty treatment, both of which developed re-restenosis (100%) during further follow-up angiography. A 3- to 5- year follow-up angiography was performed in 699 patients with 861 lesions, at a mean follow-up period of 40±9 months after stenting. The MLD increased from 0.65±0.44 mm to 3.02±0.46 mm immediately after stent implantation, decreased to 2.06±0.77 mm at the 6-month follow-up, and then increased to 2.27±0.68 mm at the 3- to 5- year angiographic follow-up (Fig. 2). There were 655 patients in the low score group, 35 patients in the intermediate score group, and 9 patients in the high score group. The long-term restenosis rate differed across the three SYNTAX score groups (5.04% in the low score group, 14.29% in the intermediate score group, and 11.11% in the high score group, p = 0.037).

**Table 4:** Quantitative Angiographic Measurements.

Patients with 6 months of follow-up	1898
No. of lesions	2364
Days to follow-up	197±60
Before stenting	
% diameter stenosis	80±13
MLD (mm)	0.63±0.43
RVD (mm)	3.12±0.47
LVEF (%)	56±14
After stenting	
% diameter stenosis	3±6
MLD (mm)	3.04±0.45
RVD (mm)	3.13±0.46
Follow-up	
% diameter stenosis	36±21
MLD (mm)	2.00±0.77
RVD (mm)	3.13±0.45
LVEF (%)	60±14
Acute gain (mm)	2.41±0.54
Late loss (mm)	1.04±0.67
Net gain (mm)	1.37±0.83
Loss index	0.45±0.30
Restenosis rate (patients)	435/1898 (23%)
Restenosis rate (lesions)	471/2364 (20%)

LVEF: left ventricular ejection fraction; MLD: minimal luminal diameter; RVD: reference vessel diameter.

### Long-term clinical outcomes

**Figure 3:**
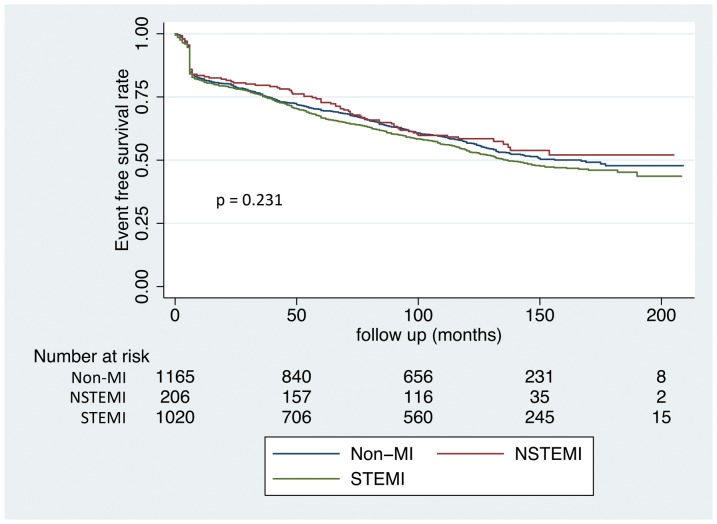
The cardiac event-free survival rate obtained by Kaplan-Meier analysis across the STEMI, NSTEMI, and non-MI groups.

During a mean follow-up period of 149±51 months, 442 patients (18.6%) died (10.8% of cardiac origin and 7.8% of non-cardiac origin), 146 (6.1%) developed reinfarction, 386 (16.2%) had TLR, 347 (14.5%) underwent stenting for new lesions, 56 (2.4%) received bypass surgery, and 115 (4.8%) developed non-fatal stroke. The overall cardiovascular event-free survival rate was 58.5%. Of the 386 patients with TLR, 311 (81%) occurred within the first year, 17 (4%) between 1 and 3 years, and 58 (15%) after 3 years. Among the 347 patients with stenting for new lesions, 45 (13%) were performed within the first year, 52 (15%) within 1–3 years, and 250 (72%) after 3 years. The total definite stent thrombosis rate was 1.6%, including 0.6% acute, 0.3% subacute, 0.3% late, and 0.4% very late stent thrombosis (Table 5). The cardiac event-free survival rate obtained from Kaplan-Meier analysis did not differ across the STEMI, NSTEMI and non-MI groups (p = 0.231) (Fig. 3). The multivariate predictors of cardiovascular events included hypertension (hazard ratio [HR]: 1.198; 95% CI: 1.011–1.421; p = 0.037), diabetes mellitus (HR: 1.277; 95% CI: 1.057–1.544; p = 0.011), the number of diseased coronary arteries (HR: 1.489; 95% CI: 1.343–1.652; p<0.001), lesion type (HR: 1.243; 95% CI: 1.110–1.392; p<0.001), and left ventricular ejection fraction (HR: 0.801; 95% CI: 0.671–0.956; p = 0.014) (Table 6). The multivariate predictors of 6-month angiographic in-stent restenosis included hypertension (HR: 1.281, 95% CI: 1.038–1.581, p = 0.037), diabetes mellitus (HR: 1.497, 95% CI: 1.195–1.876, p<0.001), lesion length (HR: 1.045, 95% CI: 1.032–1.059, p<0.001), ostial lesion (HR: 2.387, 95% CI: 1.609–3.541, p<0.001), and post-stenting MLD (HR: 0.365, 95% CI: 0.281–0.476, p<0.001) (Table 7).

**Table 5:** Clinical Events During Follow-up.

No. of patients	2379
Mortality	442(18.6%)
Cardiac	256(10.8%)
Non-cardiac	186(7.8%)
Reinfarction	146(6.1%)
Recurrent angina	510(21.4%)
Target lesion revascularization	386(16.2%)
New lesion stenting	347(14.5%)
Coronary bypass surgery	56(2.4%)
Non-fatal stroke	115(4.8%)
Cardiac event-free survival	1392(58.5%)
Stent thrombosis	38(1.6%)
Acute	14(0.6%)
Subacute	7(0.3%)
Late	8(0.3%)
Very late	9(0.4%)

**Table 6:** Multivariate Predictors of Cardiovascular Event.

	Odds Ratio	95% CI	p value
Hypertension	1.198	1.011–1.421	0.037
Diabetes mellitus	1.277	1.057–1.544	0.011
Number of diseased coronary arteries	1.489	1.343–1.652	<0.001
Lesion type	1.243	1.110–1.392	<0.001
LVEF(%)	0.801	0.671–0.956	0.014

95% CI: 95% confidence interval; LVEF: left ventricular ejection fraction.

**Table 7:** Multivariate Predictors of In-stent Restenosis.

	Odds Ratio	95% CI	p value
Hypertension	1.281	1.038–1.581	0.037
Diabetes mellitus	1.497	1.195–1.876	<0.001
Lesion length	1.045	1.032–1.059	<0.001
Ostial lesion	2.387	1.609–3.541	<0.001
Post-stenting minimal luminal diameter	0.365	0.281–0.476	<0.001

95% CI: 95% confidence interval.

## Discussion

The major findings of this study were as follows: (1) long-term (8–17 years) clinical follow-up results from a large sample size of 2379 patients who underwent BMS implantations; (2) most of the TLR cases (81%) developed within 1 year because of neointimal hyperplasia, however, most of the new lesions (72%) required stentings after 3 years because of progressive atherosclerotic changes and inflammation; (3) the long-term (3–5 years) follow-up angiographic study revealed a late increase in MLD in 699 patients and 861 lesions from the CAPTAIN registry.

Several studies with small patient populations (fewer than 1000 patients) at an earlier BMS era reported favorable clinical results after medium-term follow-up (3 years) [5]–[7], [9]. Van Domburg et al. [8] reported 10-year outcomes after BMS implantation in a single-center study including 1000 patients. During a mean follow-up period of 29 months, 8.2% of the patients died, 12.8% had non-fatal acute myocardial infarction, 13.1% underwent coronary artery bypass grafting, and 22.4% underwent repeat percutaneous transluminal coronary angioplasty. In comparison, our results showed a higher mortality rate (18.6%), and lower reinfarction (6.1%), bypass surgery (2.4%) and repeat TLR rates (16.2%) after a longer follow-up period. Yamaji et al. [11] reported very long-term (15–20 years) clinical and angiographic outcomes after coronary BMS implantation in 405 patients and demonstrated a 20.6% cardiac mortality rate, 45.5% total mortality rate, and 1.5% definite very late stent thrombosis rate at 15 years. The incidence of late TLR increased steadily from 3.3% at 4 years to 24.7% at 15 years. However, more late PCI procedures were performed for new lesions. The same authors also reported the results of a quantitative angiographic analysis of 131 patients with 179 lesions, and observed a triphasic luminal response characterized by an early restenosis phase until 6 months, an intermediate-term regression phase from 6 months to 3 years, and late renarrowing phase beyond 4 years [13]. They suggested that fibrotic scar formation associated with maturation of smooth muscle cells and reduction in matrix proteoglycans can protect the target lesions from atherosclerotic progression and plague rupture [13]. However, inflammatory reactions with prominent infiltration by lipid-laden macrophages and strong collagen-degrading matrix metalloproteinase immunoreactivity were found around the stent struts and arteries [15]. This inflammation combined with lipid-laden atherosclerotic neointima could be the underlying mechanism of late luminal re-narrowing [13]. The present study enrolled a larger patient population (699 patients with 861 lesions) who had undergone a 3- to 5- year follow-up angiography, and the results indicated the same change in luminal diameter as in the previous reports. Regarding the predictors of MACE, van Domburg et al. [8] reported multivessel stenting, stent implantation in saphenous bypass grafts, diabetes, anticoagulant treatment, bailout stenting, multivessel disease, and multiple stent implantations as predictors of MACE. They also indicated that an ejection fraction <50%, multivessel disease, diabetes, stent implantation in saphenous vein grafts, indication for unstable angina, and female sex as independent predictors of increased mortality after stenting. Another study [10] with a long-term follow-up period reported that hypertension, unstable angina, multivessel disease and multiple stent implantations were predictors of MACE, and unstable angina, lower left ventricular ejection fraction and saphenous vein graft stenting were predictors of death. The present study demonstrated similar results with regards to the risk factors of hypertension and diabetes, advanced coronary artery disease with multivessel disease and complex lesion type, and poor left ventricular ejection fraction. With regards to stent thrombosis after bare-metal stenting, Caixeta et al. [16] reported a definite or probable stent thrombosis rate of 2.0% in a pooled analysis of four randomized trials with 870 patients who underwent BMS implantations after 5 years of follow-up. Furthermore, the very late stent thrombosis rate was 0.7%. Auer et al. [17] reported a rate of angiographically confirmed stent thrombosis of 1.1% after a mean follow-up period of 3.2 years in 399 patients with BMS implantations. These findings are very similar to those of the present study, although the present study had a longer follow-up period (1.6% total definite stent thrombosis and 0.4% very late stent thrombosis rates). In addition, the predictors of 6-month angiographic restenosis in the current study included the patient-related factors of hypertension and diabetes, lesion-related characteristics of lesion length and ostial lesion, and the procedure-related factor of post-stenting MLD. All of these predictors have been reported and well-documented in previous studies [18]–[22], except for ostial lesion which have yet to be confirmed. Nevertheless, our study documented that ostial lesion was a strong independent predictor of restenosis clearly.

Regarding the angiographic pattern of in-stent restenosis, most of the restenosis lesions (82%) in this study were pattern I or II (40% and 42%, respectively), which is slightly different from those reported in previous study. Mehran et al. [14] observed 42%, 21%, 30%, and 7% of the lesions with pattern I, II, III, and IV, respectively. Moreover, conventional balloon angioplasty in the treatment of in-stent restenosis was safe and achieved good angiographic results during the follow-up periods, except in the totally occluded lesions. The restenosis rate after the balloon angioplasty was associated with the complexity of the in-stent restenosis pattern. The more complex in-stent restenosis pattern had higher restenosis rate.

### Study limitations

This study had several limitations. First, no control group was included, such as a balloon angioplasty treatment group, to demonstrate the unique benefits of stenting. Second, the rate of follow-up angiography after 3–5 years was low because of most patients were asymptomatic and declined further angiographic studies. Third, many of the factors that evolved with time influenced disease progression and the study observations, such as, interventional devices, adjunctive pharmacology and patient education. Fourth, no data regarding medications, risk factor control, and patient compliance were available, which may have also influenced the results. Fifth, the majority of the implanted BMSs are no longer used, although we can get some information from this study.

## Conclusion

This large-scale study provides clinical and angiographic results in patients who underwent BMS implantations after a long-term follow-up period (8–17 years). The cardiovascular event-free survival rate was 58.5%. The progression of coronary atherosclerosis developed over time, and presented with new lesion required stent implantation. This study included a large patient population and reconfirmed the late and sustained improvement in luminal diameter between 6 months and 3–5 years during angiographic follow-up.
